# Accuracy of the unified approach in maternally influenced traits - illustrated by a simulation study in the honey bee (*Apis mellifera*)

**DOI:** 10.1186/1471-2156-14-36

**Published:** 2013-05-06

**Authors:** Pooja Gupta, Norbert Reinsch, Andreas Spötter, Tim Conrad, Kaspar Bienefeld

**Affiliations:** 1 Institute for Bee Research Hohen Neuendorf, 16540 Hohen Neuendorf, Germany; 2 Institute of Mathematics, Freie Universitaet Berlin, Berlin, Germany; 3 Leibniz Institute for Farm Animal Biology, 18196 Dummerstorf, Germany

**Keywords:** Maternal effects, Single-step approach, Genetic evaluation, Single nucleotide polymorphism

## Abstract

**Background:**

The honey bee is an economically important species. With a rapid decline of the honey bee population, it is necessary to implement an improved genetic evaluation methodology. In this study, we investigated the applicability of the unified approach and its impact on the accuracy of estimation of breeding values for maternally influenced traits on a simulated dataset for the honey bee. Due to the limitation to the number of individuals that can be genotyped in a honey bee population, the unified approach can be an efficient strategy to increase the genetic gain and to provide a more accurate estimation of breeding values. We calculated the accuracy of estimated breeding values for two evaluation approaches, the unified approach and the traditional pedigree based approach. We analyzed the effects of different heritabilities as well as genetic correlation between direct and maternal effects on the accuracy of estimation of direct, maternal and overall breeding values (sum of maternal and direct breeding values). The genetic and reproductive biology of the honey bee was accounted for by taking into consideration characteristics such as colony structure, uncertain paternity, overlapping generations and polyandry. In addition, we used a modified numerator relationship matrix and a realistic genome for the honey bee.

**Results:**

For all values of heritability and correlation, the accuracy of overall estimated breeding values increased significantly with the unified approach. The increase in accuracy was always higher for the case when there was no correlation as compared to the case where a negative correlation existed between maternal and direct effects.

**Conclusions:**

Our study shows that the unified approach is a useful methodology for genetic evaluation in honey bees, and can contribute immensely to the improvement of traits of apicultural interest such as resistance to *Varroa* or production and behavioural traits. In particular, the study is of great interest for cases where negative correlation between maternal and direct effects and uncertain paternity exist, thus, is of relevance for other species as well. The study also provides an important framework for simulating genomic and pedigree datasets that will prove to be helpful for future studies.

## Background

A colony trait (e.g. honey and wax production) in the honey bee is comparable to maternally influenced traits in mammals such as birth and weaning weight; thus, it can be partitioned into the additive genetic effect of the queen (maternal genetic effects) and the additive genetic effects of the progeny workers (direct genetic effects). The queen mediates its effect through heritable characters like egg laying rate or pheromone production in the hive whereas workers affect a trait through their hoarding behaviour or production of and responsiveness to pheromones. Until now, genetic evaluation in the honey bee has been implemented using a pedigree based BLUP-animal model with maternal and direct genetic effects [1]. In the last decades, genetic evaluation strategies in agricultural animals have undergone remarkable advancement as a result of the introduction of genomic selection strategy. Genomic selection [2], which is based on high-density molecular marker information, has now become the ‘state of the art’ method for genetic evaluation. For many livestock species, for example, in US dairy cattle [3] and pigs [4], a multi-step procedure for genomic selection was proposed. This multi-step procedure has certain disadvantages with respect to the honey bee. Due to economical and technical constraints, it may not be possible to genotype a large number of animals in the honey bee population. Thus, instead of a multi-step procedure, we employed a single-step unified approach in our study. The unified approach was proposed by Legarra et al. [5] and Christensen and Lund [6], and it combines full pedigree and genomic information from both genotyped and ungenotyped individuals. The advantage of this procedure over the multi-step approach is that it gives a more accurate estimate of breeding values for ungenotyped animals [6,7] and is resistant to selection bias [8]. Moreover, it is simpler to implement as compared to the multi-step approach and provides an easy extension to a multi-trait model [9] with maternal effects in honey bees.

We performed a simulation study to investigate the impact of the unified approach on the accuracy of estimated breeding values in honey bees. Similar to the case of the honey bee, other species also have a situation where genetic evaluation needs to account for maternal effects and uncertain paternity, e.g. weaning weight in beef cattle is a maternally influenced trait. Besides, cows can be exposed to more than one male in a herd and pasture paddock within the same breeding season, thus generating uncertainty on paternity assignments and adversely affecting the accuracy of breeding value predictions [10]. In this study, we simultaneously dealt with the effect of uncertain paternity and maternal effects on genomic predictions. Therefore, the study is of broad interest, and can be of use for other species where maternal effects or/and uncertain paternity exists. With the exception of an abstract contribution by Lourenco et al. [11] this is, to the authors’ knowledge, the first simulation study where the application of a unified approach is evaluated for a trait with direct and maternal genetic effects.

## Methods

This study consisted of two main steps. In the first step, a dataset was simulated for a honey bee population, which involved the modelling and simulation of the population structure, genome, correlation between maternal and direct effects, heritability, true breeding values, genetic, phenotypic and residual variances. The simulated dataset was close to realistic scenarios and in agreement with the genetic and reproductive peculiarities of the honey bee. In the second step, genetic evaluation was performed using the unified approach and the traditional pedigree based BLUP approach.

### Population structure

#### Base population in linkage disequilibrium

A random mating population was simulated for 1000 generations to obtain a base population in mutation-drift equilibrium with linkage disequilibrium (LD) [12]. The simulated generations were discrete and non-overlapping. The population size was kept constant in every generation, and consisted of 500 sire queens and 50 dam queens. A sire queen represented a drone-producing queen that produced only drones whereas a dam queen represented a queen that mated with the drones to produce offspring for the next generation. The resulting base population was assumed to be non-inbred and unrelated.

#### Mating and selection scheme

**Figure 1:**
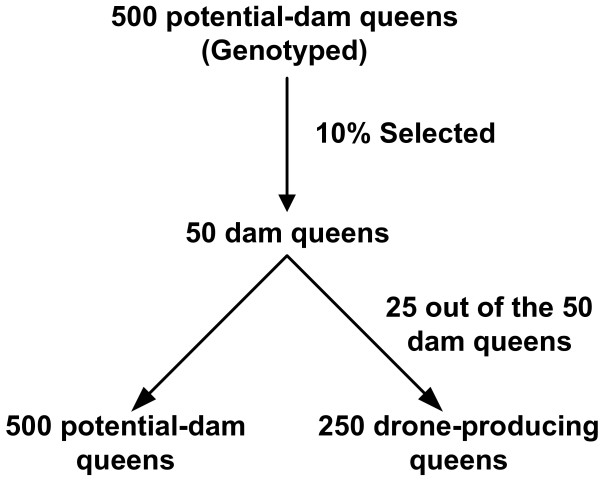
**Selection scheme.** Selection scheme showing that in each generation 10% potential-dam queens were selected randomly to serve as parent. These selected queens produced potential-dam queens and drone-producing queens for the next generation.

Five additional overlapping generations were simulated from the base population. Each of the generations consisted of 500 potential-dam queens and 250 drone-producing queens. From these 500 potential-dam queens, 10% were randomly selected as dam queens. The 50 selected dam queens produced 500 potential-dam queens (Figure 1). In addition, 25 out of the 50 selected dam queens produced 250 drone-producing queens (Figure 1). As a result, the population size in each generation remained constant with 500 potential-dam queens and 250 drone-producing queens.

#### Population characteristics specific to the honey bee

To construct a population similar to that used in the genetic evaluation program of honey bees, we constructed a dummy sire and an average worker (representing direct effects) in the pedigree. Generations following the base population were overlapping and mating was polyandrous as in the normal breeding population. These characteristics are described in more detail in the following section.

##### Construction of a dummy sire and an average worker

**Figure 2:**
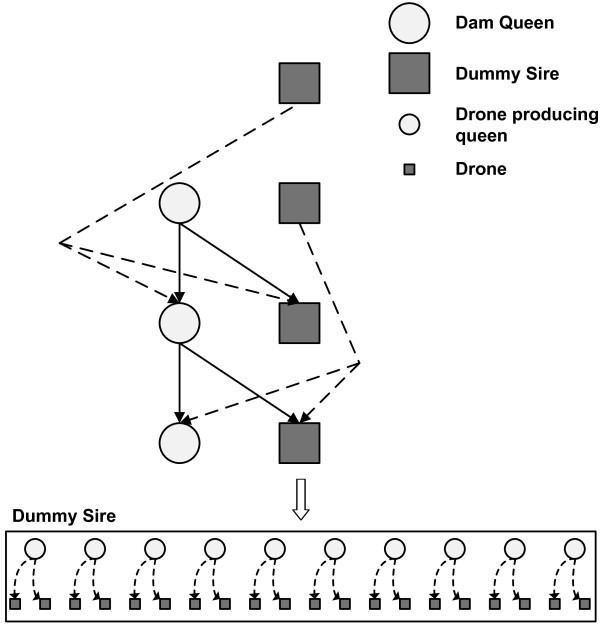
**A pedigree diagram.** In the pedigree diagram, the expanded rectangular box shows a dummy sire which consists of 10 drone-producing sister queens represented by smaller circles. Each drone-producing sister queen contributes two drones which are represented by the smaller square boxes. All drone-producing sister queens comprising a dummy sire have a common dam queen and dummy sire, thus are related as sisters. The pedigree shows that mating takes place between overlapping generations.

As a consequence of polyandry in honey bees, offspring have an unclear paternal descent. To overcome the problem of representing the paternal descent, Bienefeld et al. [1] suggested using a dummy sire in the pedigree. A dummy sire represents a group of sister colonies (approximately 8–10 sister colonies) which are maintained at the mating stations [13,14] with the purpose of producing only drones to ensure controlled mating. An example pedigree depicting a dummy sire is shown in Figure 2. For the current study, it was assumed that a dummy sire consisted of 10 drone-producing queens, thus, each generation consisted of 25 dummy sires formed by 250 drone-producing queens. It should be noted that in generations from 1–5, the 10 drone-producing queens that formed a dummy sire were related as sisters as they had the same dam queen and dummy sire (Figure 2), a situation similar to mating stations used in several countries.

A colony is formed by a queen and its progeny comprising several thousand workers. Since it is impossible to include all workers of a colony for genetic evaluation, an average worker was constructed that represented all workers of a colony. It was assumed that one average worker existed for each potential-dam queen/dam queen in the pedigree.

##### Modelling polyandry and overlapping generations

**Figure 3:**
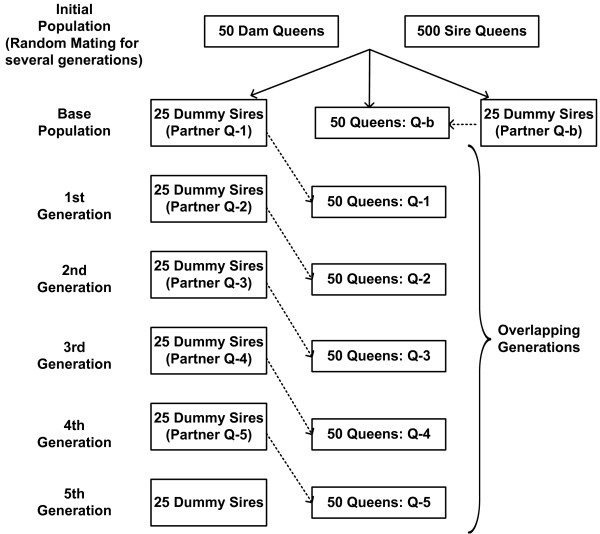
**The mating scheme.** The mating scheme is illustrated in this figure. Dummy sires connected through dashed arrow to queens are the mating partners. Q-b are queens belonging to the base population and Q-1 to Q-5 are queens belonging to generations 1–5. Each dummy sire is equivalent to 10 drone-producing sister queens. Therefore, in total, 25 dummy sires represent 250 queens. Each generation consisted of queens and a corresponding average worker for each queen to represent a colony structure, and additionally, drone-producing sister colonies in the form of dummy sire. The mating scheme shows that all generations following the base population were overlapping.

In each generation, 50 dam queens and 25 dummy sires were randomly selected as mating partners (Figure 3). A dam queen mated with one specific dummy sire, whereas a dummy sire mated with more than one dam queen. To model polyandry, each dummy sire provided 20 drones (two from each drone-producing queen) to the dam queen for mating. For generations to be overlapping, queens that were chosen to become dam queens were sampled from the n^th^ generation and queens constituting a dummy sire were taken from the (n-1)^th^ generation i.e. one generation preceding the dam queens (Figure 3). This mating scheme was consistent with the mating strategy followed by most bee breeders in several European countries. It resulted in the offspring within colonies being related as ‘super-sibs’, ‘full-sibs’ or ‘maternal half-sibs’. Super-sibs or full-sibs have a common mother and a common dummy sire. A paternal gamete comes from a single drone in case of super-sibs and different drones derived from the same queen in case of full-sibs. Maternal half-sibs also share the same mother and dummy sire, but a paternal gamete comes from different drones derived from two sister queens.

#### Pedigree, phenotypic and genomic information

A phenotypic value in the honey bee represents an observation for the whole colony and thus, cannot be decomposed into individual phenotypic values of a queen and an average worker. Therefore, both the queen and the average worker of a colony were assigned the same colony phenotypic value. It was assumed that pedigree records were available for all generations; phenotypes were available for all dam queens (and the corresponding average worker) in the base generation and all potential-dam queens (and the corresponding average worker) in all but the last generation. Genotyping information was available for all dam queens in the base generation and all potential-dam queens.

### Genome

We simulated a realistic genomic dataset which helped to assess the impact and applicability of the unified approach to the honey bee. A diploid genome consisting of 16 linkage groups was simulated for every queen [15]. A total of 100 000 loci were simulated across the genome. The length of all chromosomes and the number of marker loci per chromosome (Table 1) was simulated according to the actual chromosome length and the fraction of SNP per chromosome in the honey bee, which was obtained from analyzing the honey bee genome database [12,16,17]. We modelled both forward and backward mutation, allowing each locus to mutate from allele 1 to allele 2 and from allele 2 to allele 1. The rate of forward and backward mutation was 0.0025 per marker locus per gamete per generation [2,12]. Mutation was modelled only up to the base generation. Recombination probabilities (R) were sampled from the Haldane’s mapping function [18]. The reported recombination rate of 19 cM/Mb was used [15,19]. In the base population, 44 000 marker loci [20] with the highest minor allele frequency (MAF) were chosen (the cut-off MAF was > 5%). Out of these 44 000 marker loci, 250 with the highest MAF were taken as quantitative trait loci (QTL) and the remaining as single nucleotide polymorphisms (SNP). Thus, for the simulated genome the average distance between adjacent SNP loci was approximately 0.001 M. QTL alleles received an effect drawn from a normal distribution *N*(0, 1).

**Table 1:** Summary of the simulated chromosome lengths and number of SNP on each chromosome of the honey bee

**Chromosome**	**Chromosome length (in base-pairs)**	**Number of SNP**
Chromosome 1	29 893 408	14 137
Chromosome 2	15 549 267	6 335
Chromosome 3	13 234 341	7 119
Chromosome 4	12 718 334	5 589
Chromosome 5	14 363 272	6 330
Chromosome 6	18 472 937	7 877
Chromosome 7	13 219 345	5 973
Chromosome 8	13 546 544	6 235
Chromosome 9	11 120 453	5 578
Chromosome 10	12 965 953	5 068
Chromosome 11	14 726 556	6 957
Chromosome 12	11 902 654	5 812
Chromosome 13	10 288 499	5 082
Chromosome 14	10 253 655	4 874
Chromosome 15	10 167 229	3 879
Chromosome 16	7 207 165	3 155
Total	219 629 612	100 000

### Correlation between maternal and direct effects

Studies in honey bees ([21]; Ehrhardt and Bienefeld, unpublished results) have shown that there is a strong negative correlation between maternal (queen) and direct (worker) effects. To model this, a total of 250 QTL were simulated, out of which 86 loci controlled the direct effects, 78 pleiotropic loci controlled both the direct and maternal effects and the remaining 86 loci controlled the maternal effects. To establish a negative correlation between maternal and direct effects, signs for QTL effects for maternal and direct genetic effects were chosen opposite to each other at the pleiotropic loci. The level of negative correlation was determined by the number of pleiotropic loci. No correlation between maternal and direct effects was obtained by randomly choosing signs for QTL effects for maternal and direct effects at the pleiotropic loci. The simulated value of correlation (*r*_*qw*_) was obtained by estimating the correlation between the maternal and direct true breeding values.

### True breeding values and phenotypic values

Maternal and direct true breeding values were simulated for all dam queens of the base population and all potential-dam queens from generations 1–5. True breeding values for maternal (*TBV*_*q*_) and direct effects (*TBV*_*w*_) for a queen were calculated using the formula $\mathrm{TB}V_{q}^{i}=\sum_{j}q_{q}^{\mathrm{ij}}a^{j}$ and $\mathrm{TB}V_{w}^{i}=\sum_{k}q_{w}^{\mathrm{ik}}a^{k}$ where *TBV*_*q*_^*i*^ and *TBV*_*w*_^*i*^ are the maternal and direct true breeding values for the *i*^*th*^ queen, respectively. *q*_*q*_^*ij*^ and *q*_*w*_^*ik*^ are QTL genotypes of the *i*^*th*^ queen at the *j*^*th*^ and *k*^*th*^ QTL controlling the maternal and direct effects, respectively and has a value of 1 or −1 for the homozygous genotypes or 0 for the heterozygous genotype. *a*^*j*^ and *a*^*k*^ are allele substitution effects at the *j*^*th*^ and *k*^*th*^ QTL.

The overall true breeding value of a queen was the sum of its maternal and direct true breeding values. The phenotype of each queen was obtained by adding the overall true breeding value of a queen to a residual value drawn from a normal distribution *N*(0, *σ*_*e*_^2^). The way the value for residual variance (*σ*_*e*_^2^) was chosen is explained in the later section.

### Genetic variance

#### Variance and covariance of maternal and direct effects

Variances of maternal (*σ*_*q*_^2^) and direct (*σ*_*w*_^2^) effects were obtained by calculating the variance of the simulated maternal and direct true breeding values, respectively. The covariance between maternal and direct effects (*σ*_*qw*_) was obtained by calculating the covariance between the maternal and direct true breeding values.

#### Total genetic variance

Usually a breeding value is defined as twice the expected deviation of an individual's progeny from the mean, or twice the ‘transmitting ability’ of an individual [22]. If we consider a complete colony as ‘offspring’ of a queen, then this colony comprises a daughter (the queen) and a family of granddaughters (the workers). These offspring express 1/2 of the mother's maternal breeding value and 1/4 of the grand-dam's direct breeding value. In this case, the overall true or estimated breeding value would be defined as twice the 1/2 of the maternal breeding value of a queen plus twice the 1/4 of its direct breeding value (i.e. two times the expected deviation of ‘progeny’ from the mean, provided all other relatives have average breeding values of zero). Thus, maternal and direct breeding values get a weight of 1 and 0.5, respectively. The total genetic variance (*σ*_*g*_^2^) would become *σ*_*q*_^2^ + 0.25*σ*_*w*_^2^ + *σ*_*qw*_ (the latter from 2 × 1 × 0.5 × *σ*_*qw*_). However, for the sake of easy comparison and interpretation, the overall breeding value was taken as a sum of the direct and maternal breeding values of a queen and the total genetic variance was taken as a sum of variance of maternal effects, direct effects and twice the covariance between them, and can be expressed as *σ*_*g*_^2^ = *σ*_*q*_^2^ + *σ*_*w*_^2^ + 2*σ*_*qw*_.

### Phenotypic variance, residual variance and maternal and direct heritability

A colony trait in honey bees is determined by the heritability of maternal (*h*_*m*_^2^) and direct (*h*_*d*_^2^) effects. In our study, we simulated a fixed maternal heritability of 0.15, 0.25 and 0.35 (e.g. honey yield, hygienic behaviour) that can be expressed as a ratio of the variance of maternal effects to the phenotypic variance and is given as follows:

$$\begin{array}{l} h_{m}^{2}=\frac{\mathrm{Variance}\;\mathrm{of}\;\mathrm{maternal}\;\mathrm{effects}}{\mathrm{Phenotypic}\;\mathrm{variance}}\;=\frac{\sigma_{q}^{2}}{\sigma_{e}^{2}+\sigma_{g}^{2}}\\ \;=\frac{\sigma_{q}^{2}}{\sigma_{p}^{2}} \end{array}$$

After rearranging, we get, $\sigma_{p}^{2}=\frac{\sigma_{q}^{2}}{h_{m}^{2}}$. Thus, for a fixed value of maternal heritability, the phenotypic variance (*σ*_*p*_^2^) was obtained from the expression $\frac{\sigma_{q}^{2}}{h_{m}^{2}}$. The residual variance (*σ*_*e*_^2^) was obtained by subtracting the total genetic variance (*σ*_*g*_^2^) from the phenotypic variance (*σ*_*p*_^2^) i.e. *σ*_*e*_^2^ = *σ*_*p*_^2^ − *σ*_*g*_^2^. The ratio of variance of direct effects to the phenotypic variance provided a measure of the heritability of direct effects, as given below.

$$h_{d}^{2}=\frac{\mathrm{Variance}\;\mathrm{of}\;\mathrm{direct}\;\mathrm{effects}}{\mathrm{Phenotypic}\;\mathrm{variance}}\;=\frac{\sigma_{w}^{2}}{\sigma_{e}^{2}+\sigma_{g}^{2}}=\frac{\sigma_{w}^{2}}{\sigma_{p}^{2}}$$

Table 2 shows the values of simulated maternal heritability and achieved direct heritability at different correlations between maternal and direct effects.

**Table 2:** Heritability of direct effects for different values of simulated heritability of maternal effects and correlation between maternal and direct effects

**Simulated*h***_***m***_^**2**^	**Corr**_**(m,d)**_	**Achieved*h***_***d***_^**2**^**(SE)**
0.150	0	0.162 (0.005)
0.150	−0.46	0.155 (0.005)
0.250	0	0.270 (0.008)
0.250	−0.46	0.259 (0.008)
0.350	0	0.377 (0.011)
0.350	−0.46	0.362 (0.011)

*h*_*m*_^2^, Maternal heritability; *h*_*d*_^2^, Direct heritability; *Corr*_*(m,d)*_, Correlation between maternal and direct effects; *SE*, Standard error.

### Estimation of breeding values

A BLUP-animal model with maternal and direct effects [23], with a numerator relationship matrix adapted to the peculiarity of the honey bee, was used for genetic evaluation [1] and is given as:

$$y=\mathrm{Xb}+Z_{1}u_{1}+Z_{2}u_{2}+e$$

where **y** is a vector of records of the colonies, **b** is a vector of fixed effects, **u**_**1**_ is a vector of random direct effects, **u**_**2**_ is a vector of random maternal effects, **e** is a vector of random residual effects, **X** is an incidence matrix relating observations to the corresponding environment, **Z**_**1**_ and **Z**_**2**_ are the incidence matrices relating observations to the corresponding direct effects and maternal effects, respectively.

Estimation of breeding values was done using the following two approaches: (1) the traditional BLUP approach (PED_BLUP) based on a numerator relationship matrix (**A**) constructed from pedigree information and (2) the unified approach (UNI_BLUP) based on a combined relationship matrix (**H**) constructed from pedigree and genomic information.

#### Relationship matrix constructed from pedigree data

Elements of the numerator relationship matrix (**A**) were calculated according to the method proposed by Bienefeld et al. [1] for honey bees which includes a paternal path coefficient (*P*_*p*_) of 0.367 to account for polyandry. This value is currently used for Germany-wide genetic evaluation of the honey bee populations where all mating sites are managed according to unified guidelines (with respect to number of drone-producing colonies and their relationship). The details for constructing the **A** matrix recursively are given in the Additional file 1. We constructed the **A** matrix for all 5275 individuals in the pedigree. The **A** matrix was partitioned into **A**_**11**_, **A**_**22**_, **A**_**12**_ and **A**_**21**_ where subscripts 1 and 2 represent genotyped and non-genotyped individuals, respectively. The inverse of the partitioned **A** matrix can be expressed as [6]:

$$A^{-1}=\left[\begin{array}{cc} A_{11}^{-1}+A_{11}^{-1}A_{12}{\left(A_{22}-A_{21}A_{11}^{-1}A_{12}\right)}^{-1}A_{21}A_{11}^{-1} & -A_{11}^{-1}A_{12}{\left(A_{22}-A_{21}A_{11}^{-1}A_{12}\right)}^{-1}\\ -{\left(A_{22}-A_{21}A_{11}^{-1}A_{12}\right)}^{-1}A_{21}A_{11}^{-1} & {\left(A_{22}-A_{21}A_{11}^{-1}A_{12}\right)}^{-1} \end{array}\right]$$

#### Relationship matrix constructed from pedigree and genomic data

In the honey bee pedigree, a dummy sire and an average worker represent a group of individuals and thus, it is not possible to get individual genotyping data. Moreover, it is not possible to obtain genotyping information from all queens in the population. Using the unified approach is advantageous for honey bees as genomic information for genotyped queens can be integrated with pedigree information from genotyped as well as non-genotyped individuals resulting in a combined relationship matrix **H**. A genomic matrix (**G**) was constructed for the 2550 queens with genotyping data. Different methods have been developed to derive the **G** matrix [24,25]. We chose a methodology proposed by VanRaden [24]. The **G** matrix was obtained from **ZZ**’/2 ∑ *p*_*i*_(1 − *p*_*i*_), where **Z** is equal to **M** − **P**, **M** is the matrix specifying marker alleles inherited by each individual and **P** is equal to 2(*p*_*i*_ − 0.5) with *p*_*i*_ being the frequency of second allele at locus *i* in the base population. In order to avoid a singular **G** matrix [5,7,24], a modified matrix (**G**_**w**_) was constructed using a weighing factor (*w*), given as **G**_**w**_ = *w***G** + (1 − *w*)**A**_**11**_. Christensen and Lund [6] suggested that (1 − *w*) could be interpreted as the relative weight on the polygenic effect. Aguilar et al. [7] reported that the weights were not critical, and using a value of 0.95 or 0.98 caused negligible difference in the results. For this study, the value of *w* was taken as 0.99 [6].

The inverse of the combined relationship matrix (**H**^-1^), described by Legarra et al. [5], Christensen and Lund [6] and Aguilar et al. [7], was computed and is given as shown below.

$$H^{-1}=A^{-1}+\left[\begin{array}{cc} G_{w}^{-1}-A_{11}^{-1} & 0\\ 0 & 0 \end{array}\right]$$

Simulated values for the genetic and residual variance were used for estimating the breeding values. For both approaches, statistics for the achieved heritability of direct effects and accuracies for the overall, maternal and direct estimated breeding values were based on 20 replicated simulations. The accuracy was reported as a correlation between the estimated and true breeding values [26] for 500 ‘juvenile queens’ constituted by potential-dam queens in the last generation and 2550 ‘all queens’ constituted by dam queens in the base population and potential-dam queens in all generations. All calculations were performed in MATLAB.

## Results

### Accuracy of the overall estimated breeding values

In the honey bee breeding programs, the criterion used for selecting queens is its overall breeding value which is a sum of the maternal and direct estimated breeding values. Therefore, in this study we report the accuracy of overall estimated breeding values. Table 3 shows the accuracy achieved for overall estimated breeding values with the UNI_BLUP and the PED_BLUP approaches.

**Table 3:** The accuracy of overall EBV (the sum of maternal and direct breeding values) in case of 500 juvenile queens and all 2550 queens in the pedigree

***h***_***m***_^**2**^	**Method**	**Corr**_**(m,d)**_	**Accuracy for JQ (SE)**	**Accuracy for AQ (SE)**
**0.15**	UNI	0	0.468 ^a,b,c,d^ (0.010)	0.661 ^a,b,c,d^ (0.005)
	PED	0	0.363 (0.017)	0.603 (0.007)
	UNI	−0.46	0.381 ^a,b,c,d^ (0.021)	0.555 ^a,b,c,d^ (0.010)
	PED	−0.46	0.295 (0.023)	0.489 (0.009)
**0.25**	UNI	0	0.542 ^a,b,c,e^ (0.009)	0.756 ^a,b,c,e^ (0.006)
	PED	0	0.420 (0.015)	0.710 (0.008)
	UNI	−0.46	0.449 ^a,b,c^ (0.018)	0.640 ^a,b,c,e^ (0.009)
	PED	−0.46	0.348 (0.021)	0.577 (0.008)
**0.35**	UNI	0	0.604 ^a,b,d,e^ (0.009)	0.832 ^a,b,d,e^ (0.008)
	PED	0	0.467 (0.012)	0.800 (0.010)
	UNI	−0.46	0.498 ^a,b,d^ (0.017)	0.700 ^a,b,d,e^ (0.008)
	PED	−0.46	0.388 (0.019)	0.642 (0.008)

*EBV*, Estimated breeding values; *h*_*m*_^2^, Maternal heritability; *UNI*, UNI_BLUP; *PED*, PED_BLUP; *Corr*_*(m,d)*_, Correlation between maternal and direct effects; *JQ*, Juvenile queens; *AQ*, All queens; *SE*, Standard error.

Significant difference in accuracy with P < 0.05 (Welch’s *t*-test) between: ^a^UNI_BLUP and PED_BLUP; ^b^no correlation and negative correlation for UNI_BLUP; ^c^heritabilities 0.15 and 0.25 for UNI_BLUP; ^d^heritabilities 0.15 and 0.35 for UNI_BLUP; ^e^heritabilities 0.25 and 0.35 for UNI_BLUP.

For juvenile queens, the accuracy of overall estimated breeding values was significantly higher with the UNI_BLUP approach (P < 0.05) as compared to the PED_BLUP approach for all values of heritability and correlation between maternal and direct effects. The increase in accuracy by UNI_BLUP was approximately 0.1 (or 29%) for most of the cases.

Similar to juvenile queens, the accuracy of overall estimated breeding values for all queens was higher with the UNI_BLUP approach (P < 0.05) than the PED_BLUP for all values of heritability and correlation between maternal and direct effects. The percentage increase in accuracy for the case of no correlation between maternal and direct effects at maternal heritabilities of 0.15, 0.25 and 0.35 was approximately 9.6%, 6.5% and 4.0%, respectively. In case of a negative correlation of −0.46, the percentage increase in accuracy was approximately 13.5%, 10.9% and 9.0% at maternal heritabilities of 0.15, 0.25 and 0.35, respectively.

From these results we can conclude that the UNI_BLUP approach performed better than the PED_BLUP and the accuracy of overall estimated breeding values increased considerably with the UNI_BLUP approach.

### Accuracy of the maternal and direct estimated breeding values

Table 4 shows the accuracy of maternal and direct estimated breeding values for juvenile queens and for all queens. The average value (over 20 replicates) of the accuracy of maternal as well as direct estimated breeding values was higher for the UNI_BLUP approach as compared to the PED_BLUP approach for all values of heritability and correlation between maternal and direct effects. However, the difference between UNI_BLUP and PED_BLUP approaches were not significant for some cases (Table 4). Overall, the accuracy of maternal and direct estimated breeding values showed a trend in favour of the UNI_BLUP approach.

**Table 4:** The accuracy of direct and maternal EBV in case of 500 juvenile queens and 2550 all queens in the pedigree

***h***_***m***_^**2**^	**Method**	**Corr**_**(m,d)**_	**Accuracy of direct EBV for JQ (SE)**	**Accuracy of maternal EBV for JQ (SE)**	**Accuracy of direct EBV for AQ (SE)**	**Accuracy of maternal EBV for AQ (SE)**
**0.15**	UNI	0	0.323 ^a,b,c,d^ (0.015)	0.279 ^a,b,c,d^ (0.015)	0.446 ^a,b,c,d^ (0.011)	0.420 ^a,b,c,d^ (0.009)
	PED	0	0.227 (0.023)	0.225 (0.019)	0.406 (0.012)	0.381 (0.010)
	UNI	−0.46	0.115 ^b,d^ (0.023)	0.127 ^b^ (0.031)	0.225 ^b,d^ (0.018)	0.223 ^b,d^ (0.013)
	PED	−0.46	0.059 (0.025)	0.103 (0.030)	0.186 (0.016)	0.208 (0.012)
**0.25**	UNI	0	0.373 ^a,b,c^ (0.016)	0.330 ^a,b,c,e^ (0.014)	0.510 ^b,c,e^ (0.013)	0.482 ^a,b,c,e^ (0.010)
	PED	0	0.268 (0.021)	0.260 (0.018)	0.474 (0.013)	0.447 (0.011)
	UNI	−0.46	0.154 ^b^ (0.023)	0.154 ^b^ (0.031)	0.272 ^b^ (0.017)	0.257 ^b^ (0.014)
	PED	−0.46	0.085 (0.025)	0.125 (0.030)	0.231 (0.016)	0.240 (0.013)
**0.35**	UNI	0	0.418 ^a,b,d^ (0.017)	0.371 ^a,b,d,e^ (0.014)	0.566 ^b,d,e^ (0.015)	0.527 ^b,d,e^ (0.011)
	PED	0	0.307 (0.018)	0.287 (0.017)	0.538 (0.015)	0.496 (0.013)
	UNI	−0.46	0.186 ^a,b,d^ (0.024)	0.173 ^b^ (0.031)	0.308 ^b,d^ (0.017)	0.280 ^b,d^ (0.015)
	PED	−0.46	0.110 (0.025)	0.138 (0.030)	0.268 (0.016)	0.258 (0.014)

*EBV*, Estimated breeding values; *h*_*m*_^2^, Maternal heritability; *UNI*, UNI_BLUP; *PED*, PED_BLUP; *Corr*_*(m,d)*_, Correlation between maternal and direct effects; *JQ*, Juvenile queens; *AQ*, All queens; *SE*, Standard error.

Significant difference in accuracy with P < 0.05 (Welch’s *t*-test) between: ^a^UNI_BLUP and PED_BLUP; ^b^no correlation and negative correlation for UNI_BLUP; ^c^heritabilities 0.15 and 0.25 for UNI_BLUP; ^d^heritabilities 0.15 and 0.35 for UNI_BLUP; ^e^heritabilities 0.25 and 0.35 for UNI_BLUP.

### Effect of correlation and heritability

Both low heritability and negative correlation contribute to a lower genetic variance which leads to a decrease in the accuracy. The accuracy of overall estimated breeding values was reduced as a result of negative correlation in comparison to the case where maternal and genetic effects had no correlation (Table 3; P < 0.05). Similarly, the accuracy of overall estimated breeding values increased as the heritability increased (Table 3; P < 0.05). The only exception, where no significant difference was observed, was between maternal heritabilities of 0.25 and 0.35 at a negative correlation of −0.46 for juvenile queens, although the accuracy was higher for high heritability. This can be explained by the fact that the impact of using genomic information is smaller for traits with high heritablities.

The accuracy of maternal and direct estimated breeding values (Table 4) was higher for high values of heritability indicating a similar trend as the overall estimated breeding values. The difference was significant in most cases for any two compared values of heritability with no correlation and between heritability of 0.15 and 0.35 with negative correlation.

## Discussion

The study provided comparative insight into genetic evaluation performed using: (1) the traditional BLUP approach based on pedigree data and (2) the unified approach based on both pedigree and marker data. In this study, we investigated the accuracy of overall, direct and maternal estimated breeding values as well as the influence of heritability of the trait and the genetic correlation between maternal and direct effects on the accuracy.

It has been reported in honey bees that most economically important traits have low to medium heritability [27-29]. Therefore, we also simulated heritabilities in the same range. The extremely negative estimates of genetic correlation between maternal and direct effects have often been questioned ([30]; Ehrhardt and Bienefeld, unpublished results), therefore, we simulated a general value of correlation of −0.46 which exists in other species as well [31-33] and compared it to a case with no correlation between maternal and direct effects.

Unlike previous studies [6,7,34], our study takes into account the influence of maternal and direct effects. We observed that the accuracy of overall estimated breeding values (Table 3) increased considerably with the unified approach for all scenarios of heritability and correlation with significant P-values (< 0.05). A higher gain in the accuracy of overall estimated breeding values was observed for juvenile animals. It is desired that the gain in accuracy is higher for juvenile animals as they are the subsequent candidates for selection. This may consequently help speeding up the selection procedure as a result of reduction in the generation interval. Similar gain in accuracy was reported in other studies. For example, in another study [34], the accuracy of estimated breeding values for genotyped female pigs was reported to be 0.22 with the pedigree based approach whereas it ranged from 0.28 to 0.49 with the unified approach depending on the **G** matrix. Likewise, Christensen and Lund [6] reported an accuracy of 0.66 with the one-step unified approach and 0.35 with the pedigree based approach.

In our study, the accuracies of maternal and direct estimated breeding values for the pedigree based approach (PED_BLUP) with maternal and direct heritability of 0.15 were 0.38 and 0.41 at no correlation and 0.21 and 0.19 at a correlation of −0.46, respectively. In an earlier pedigree based study by Roehe and Kennedy [35], the accuracy of maternal and direct estimated breeding values was reported to be 0.21 (0.21) and 0.38 (0.28) for the case of no correlation and 0.19 (0.18) and 0.31 (0.23) for a negative correlation of −0.5 in female (male) pigs for maternal and direct heritability of 0.05 and 0.1, respectively. These estimates were based on a pedigree based complete animal model with maternal effects. The difference in the accuracies between our study and to that reported by Roehe and Kennedy [35] can be a result of dissimilarities between the two studies such as the construction of the numerator relationship matrix, value of simulated maternal and direct heritability, random selection of the individuals, number of generations simulated, population structure and size. Nonetheless, the comparison of results of the pedigree based approach with the study from Roehe and Kennedy [35] helps to assess and validate the values of accuracy of maternal and direct estimated breeding values obtained in our study. In our study, the accuracies of maternal and direct estimated breeding values were higher for the UNI_BLUP approach as compared to the PED_BLUP approach, but the difference between UNI_BLUP and PED_BLUP approaches were not significant for some cases (Table 4). Thus, in order to achieve maximum gain from implementing the unified approach, a proper investigation into the cost benefits and the relative improvement in genetic gain is required for traits selected solely on the basis of maternal or direct breeding values. Nevertheless, the sum of maternal and direct effects is still the most important criterion for selection and the use of only direct or maternal effects is not helpful for the honey bee.

A complexity associated with the estimation of breeding values for maternally influenced traits is that negative correlation between maternal and direct effects severely impedes the response to selection [35,36]. Additionally, it leads to a decrease in the total genetic variance resulting in lowered accuracies. As shown in the results, the accuracy of estimated breeding values improved significantly in case of negative correlation with the unified approach as compared to the pedigree based approach. We propose that the extra gain from genomic selection (versus pedigree-BLUP) is larger, when the correlations between direct and maternal effects are negative, compared to scenarios with positive correlations. This is because some of the markers capture maternal (direct) genetic differences, induced by loci without pleiotropic direct (maternal) effects. These parts of the genetic variation are of special interest in case of negative correlations between direct and maternal effects, since they provide, at least partially, opportunity for achieving both maternal and direct genetic gain in the desired direction. In contrast to this, each positive maternal (direct) gain stemming from pleiotropic loci is counterbalanced by a negative direct (maternal) one. Obviously, this kind of distinction does not matter at all in case of positive correlations and is even impossible to be exploited with only pedigree information at hand. So, genomic selection offers special advantages in cases, where maternal and direct effects are negatively correlated through the pleiotropic action of common loci, a phenomenon probably deserving more attention and research also in other species. Additionally, the increase in accuracies can be attributed to the genomic matrix which is able to provide a more precise measure of genetic relatedness. The numerator relationship matrix uses pedigree information to derive the probability of genes to be identical by descent that gives an estimate of the relatedness of individuals. The genomic matrix, in contrast, uses high-density marker information and thus, can identify genes that are identical by state and may be shared through common ancestor not recorded in the pedigree [34]. Hence, it provides a more accurate measure for the relationship between individuals. It also enables better differentiation among closely related individuals since it captures Mendelian sampling with greater precision. Thus, the use of a marker based relationship matrix in the unified approach greatly helps to improve the accuracy of estimated breeding values for low heritability traits and/or negatively correlated traits, e.g. traits with negatively correlated maternal and direct effects.

## Conclusions

To provide a comparison between genetic evaluation methods based on the unified approach and the pedigree based approach, we modelled a complex scenario by taking into consideration characteristics such as varying heritability and correlation between maternal and direct genetic effects, uncertain paternity and other genetic and reproductive peculiarities of the honey bee. To the best of our knowledge, this is the first study that describes the use of molecular marker data for genetic evaluation in honey bees by employing the unified approach. The study provides background knowledge about the simulation of a genomic and a pedigree dataset in honey bees for genetic evaluation, therefore, it can serve as an important framework for future studies. Studies in other species [7,34] have already optimized the approach with respect to the construction of genomic matrix and computational solving procedures. Thus, additional investigation is needed in future to improve the methodology in the honey bee. The unified approach is a progressive step in the genetic evaluation program of honey bees that will facilitate to reduce the rates of inbreeding, improve the genetic gain and response to selection.

## Abbreviations

BLUP: Best Linear Unbiased Prediction; LD: Linkage Disequilibrium; MAF: Minor Allele Frequency; QTL: Quantitative Trait Loci; SNP: Single Nucleotide Polymorphism

## Competing interest

The authors declare that they have no competing interests.

## Authors’ contributions

PG conducted the study and wrote the manuscript. NR and KB conceived the study, participated in discussions and helped to draft the manuscript. AS and TC participated in discussions and helped to draft the manuscript. All authors read and approved the final manuscript.

## Supplementary Material

Additional file 1 Details for constructing the honey bee’s numerator relationship matrix recursively. Click here for file
