# Supplementary material for: Accuracy of the unified approach in maternally influenced traits - illustrated by a simulation study in the honey bee (Apis mellifera)
Source: BMC Genet. 2013 May 6;14:36. doi: 10.1186/1471-2156-14-36 (PMC3654974; doi:10.1186/1471-2156-14-36)
Supplement: Additional file 1 — Details for constructing the honey bee’s numerator relationship matrix recursively. [file 1471-2156-14-36-S1.pdf]

Additional file 1

"Accuracy of the unified approach in maternally influenced traits - illustrated by a simulation study in the honey bee (*Apis mellifera*)". Manuscript Id: 1014723498670742

Gupta, P., Reinsch, N., Spötter, A., Conrad, T., and Bienefeld, K.

Details for constructing the honey bee's numerator relationship matrix recursively

If both sire and dam are known

$$a_{ji} = a_{ij} = 0.5a_{jd} + P_p(a_{js}) \qquad for\ \ j = 1\ to\ (i-1)$$

$$a_{ii} = 1 + 0.5(a_{sd})$$

If only sire is known and assumed to be unrelated to the dam

$$a_{ji} = a_{ij} = P_p(a_{js}) \qquad for\ \ j = 1\ to\ (i-1)$$

$$a_{ii} = 1$$

If only dam is known and assumed to be unrelated to the sire

$$a_{ji} = a_{ij} = 0.5a_{jd} \qquad for\ \ j = 1\ to\ (i-1)$$

$$a_{ii} = 1$$

If both parents are unknown and assumed to be unrelated

$$a_{ji} = a_{ij} = 0 \qquad for\ \ j = 1\ to\ (i-1)$$

$$a_{ii} = 1$$
